# Systematic Review of the Role of BCG in the Treatment of Urothelial Carcinoma of the Prostatic Urethra

**DOI:** 10.3233/BLC-201516

**Published:** 2021-05-25

**Authors:** Oliver Patschan, Philippe E. Spiess, George N. Thalmann, Joan Palou Redorta, Georgios Gakis

**Affiliations:** a Institution of Translational Medicine, Lund University, Malmö, Sweden; b Department of GU Oncology and Department of Tumor Biology, Moffitt Cancer Center, Tampa, FL, USA; c Department of Urology, University Hospital Inselspital, Bern, Switzerland; d Department of Urology, Hospital de la Santa Creu i Sant Pau, Universitat Autonoma de Barcelona, Fundatió Puigvert, Barcelona, Spain; e Department of Urology and Pediatric Urology, University Hospital of Würzburg, Würzburg, Germany

**Keywords:** BCG, transitional carcinoma, prostatic urethra

## Abstract

**BACKGROUND:**

In patients with non-invasive urothelial carcinoma of the prostatic urethra (PUC), treatment with Bacillus Calmette-Guérin (BCG) could be beneficial.

**OBJECTIVE:**

To assess the response rates to BCG in the different tumor stages, to describe the clinical impact of transurethral resection of the prostate (TURP) before BCG treatment, and to review the side effects of BCG treatment for PUC.

**METHODS:**

A systematic search was conducted using the PubMed database to identify original studies between 1977 and 2019 reporting on PUC and BCG.

**RESULTS:**

Of a total of 865 studies, ten were considered for evidence synthesis. An indication for BCG treatment was found in non-stromal invasive stages (Tis pu, Tis pd) and in stromal infiltrating cases (T1) of primary and secondary PUC when transitional cell carcinoma was the histology of origin. Studies including patients treated with TURP before BCG showed a better local response in the prostatic urethra with a higher disease free survival (DFS) (80–100% vs. 63–89%) and progression free survival (PFS) (90–100% vs. 75–94%) than patients in studies in which no TURP was performed. However, this difference in recurrence and progression in the prostate neither affected the total PFS (57–75% vs. 58–93%), nor the disease specific survival (70–100% vs. 66–100%).

**CONCLUSIONS:**

The use of resection loop biopsies of the prostatic urethra in appropriate cases during the primary work-up for suspected PUC, as well as the use of the current TNM classification for PUC, need to be improved. BCG therapy for non-stromal invasive stages of PUC show a good local response. Local response is further improved by a TURP before BCG therapy, although the overall prognosis does not seem to be affected. Further evidence for BCG treatment in the rare cases of stromal invasive PUC is needed. Specific side effects of BCG treatment for PUC are not reported.

## INTRODUCTION

Urethral carcinoma is a rare disease, accounting for less than 1% of all urogenital cancer cases [1]. In the 28 European Union countries, it is estimated that 655 patients are being diagnosed with urethral carcinoma annually, striking patients from the fifth decade of life and with a peak incidence in patients 75 years of age [2, 3]. Primary urethral carcinoma is detected in patients without a previous history of urothelial cancer. Secondary urethral carcinoma occurs during the follow up of a known urothelial cancer of the bladder or the upper urinary tract (UUT).

The histological origin of urethral carcinoma differs between the sexes: In women, adenocarcinoma is the most frequent histology (38–47%) followed by SCC (25–28%), UC (25–28%) and other histological entities (6%) [4, 5]. In women, both surgery and radiation therapy are practical treatment options [6]. In men, approximately 80% of the cases of urethral carcinoma are urothelial cancers, followed by squamous cell carcinoma (15%) and adenocarcinoma (5%), respectively [7]. The histologic features of these cancers vary by anatomical location: Urethral cancers in the penile or bulbar urethra are of squamous cell differentiation in about 90% of the cases. In contrast, in the prostatic urethra, 90% of the cancers are of urothelial origin [8]. Generally, the primary treatment of male urethral carcinoma is surgical excision. In the prostatic urethra, transurethral resection is often the first diagnostic and therapeutic step. In invasive growth of prostatic urethral carcinoma (PUC), radical cystoprostaturethrectomy, either before or after chemotherapy, is indicated.

Bacillus Calmette-Guérin (BCG) is the currently most potent drug for intravesical treatment of high-risk non-muscle invasive bladder cancer (NMIBC). It is known to improve intravesical recurrence-free survival and might even improve progression-free survival in non-muscle invasive bladder cancer [9]. Since PUC originates almost exclusively from the urothelium, BCG is a relevant drug even for the treatment of urethral carcinoma of the prostatic urethra. In patients without stromal invasion (Tis pu, Tis pd) and even with stromal invasion (T1) of urothelial carcinoma of the prostatic urethra, treatment with BCG might be beneficial [6]. However, the evidence for BCG treatment, with or without transurethral resection of the prostate (TURP) before treatment induction, has been sparse. This systematic review aims to elucidate the clinical impact of BCG treatment in the different stages of urothelial carcinoma of the prostate. It also aims to assess the effect of TURP before BCG treatment on the risk for recurrence and progression, and to review the side effects of BCG treatment for PUC.

## EVIDENCE ACQUISITION

A systematic review was carried out based on a literature search by PubMed/Medline. Due to the low incidence of the disease, no prospective clinical trials on BCG treatment in PUC were available. This explains why a systematic review according to the PICO description was not possible. All authors participated in the process of literature search and data acquisition process.

This literature search was aimed at identifying all articles that published the results of cohort studies and retrospective clinical studies as full-length articles published in English between 1977 (date of the first publications relative to urethral carcinoma) and March 2019. Case reports and reviews were excluded.

The following keywords were used in the database just cited: Bacillus Calmette-Guérin OR BCG AND urethral carcinoma OR urothelial cancer or prostatic urethra; carcinoma in situ OR CIS AND urethral carcinoma OR urothelial cancer prostatic urethra; radical cystectomy AND urethral carcinoma OR urothelial cancer prostatic urethra; Bacillus Calmette-Guérin OR BCG AND urethral carcinoma OR urothelial cancer prostatic urethra AND survival; Bacillus Calmette-Guérin OR BCG AND urethral carcinoma OR urothelial cancer prostatic urethra AND transurethral resection prostate; Bacillus Calmette-Guérin OR BCG AND urethral carcinoma OR urothelial cancer prostatic urethra AND radical cystectomy; Bacillus Calmette-Guérin OR BCG AND urethral carcinoma OR urothelial cancer prostatic urethra AND side effects.

The reference lists of all systematic reviews in the field were screened for additional references. After a first selection, based on the title and abstract of the papers, duplicates were removed. Once selected, the full text of the articles was studied to gather information about study design, inclusion criteria, baseline patient characteristics, TNM-stage, treatment regimen, follow up, disease free survival (DFS), progression free survival (PFS), and disease specific survival (DSS).

## EVIDENCE SYNTHESIS

### BCG-response in the different tumor stages of prostatic urethral carcinoma

**Fig. 1:**
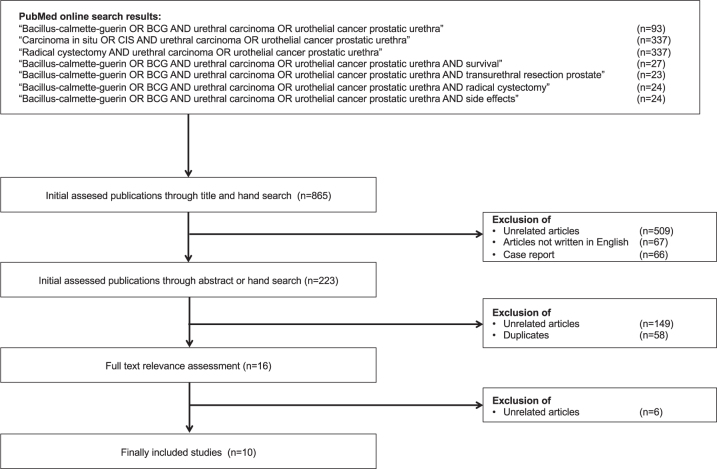
Consolidated Standards of Reporting Trials diagram outlining the selection process of the included studies. Date of search: 30th september 2019.

BCG treatment was reported in two clinical scenarios of PUC: Primary and secondary urothelial carcinomas of the prostatic urethra [10–19]. No articles were found describing BCG treatment in patients with primary urethral carcinoma of the anterior urethra. There were also no articles specifically addressing primary cases of PUC without concomitant carcinoma of the urinary bladder or the upper urinary tract. Figure 1 shows the selection process for the included articles. A total of ten retrospective studies focused on patient outcomes in patients with PUC treated with BCG. A total of 162 patients were included in the studies on this topic, of which only 5 patients were shown to have tumor stage T1. The study by Taylor et al. [15] is an update of the study by Schellhammer et al. [18], which in turn is an update of the study by Hillyard et al. [19], and it cannot be ruled out that some of the patients included in these studies were examined twice. BCG was used only in superficial stages of PUC (Tis pu, Tis pd, T1). The TNM classification of these tumor stages is shown in Table 1 [20]. Data from all selected studies are shown in Table 2.

**Table 1:** TNM classification (eighth edition) for urethral carcinoma. Non-muscle invasive stages of urothelial carcinoma of the prostatic urethra only [20]

Tis pu	Carcinoma in situ, involvement of prostatic urethra
Tis pd	Carcinoma in situ, involvement of prostatic ducts
T1	Tumor invades subepithelial connective tissue (fortumors involving prostatic urethra only)

Urothelial (transitional cell) carcinoma of the prostate.

**Table 2:** Follow-up studies on patients with different stages of non-invasive urothelial carcinoma of the prostatic urethra treated with or without TURP before BCG treatment. Tumor progression was defined by the development of muscle infiltration, metastatic disease or the occurrence of superficial disease refractory to transurethral resection and intravesical BCG necessitating a change in therapy. Survival data in relation to the median follow-up time of the cited study. Abbreviations: TURP = transurethral resection of the prostate; TURP = transurethral resection of the prostate; BCG = Bacillus Calmette Guérin; DFS = disease free survival; DSS = disease specific survival (i.e. disease specific cause was including urothelial cancer in the bladder or the upper urinary tract); PFS = progression free survival; N.A.  = not available/not applicable

Reference	+/- TURP before BCG	Total number of prostatic urethral cancer Patients treated with BCG	Primary prostatic urethral cancer cases n/N (%)	Mucosal prostatic urethral cancer (Tis pu) n/N (%)	Ductal prostatic urethral cancer (Tis pd) n/N (%)	Stromal prostatic urethral cancer (T1) n/N (%)	BCG regimen	BCG strain	Median follow up in months (range)	Bladder cancer DFS n/N (%)	Bladder cancer PFS n/N (%)	Prostatic urethral cancer DFS n/N (%)	Prostatic urethral cancer PFS n/N (%)	Prostatic urethral+bladder cancer DFS n/N (%)	Prostatic urethral+bladder cancer PFS n/N (%)	DSS n/N (%)
Gofrit et al. (2008) [10]	+	20	16/20 (80%)	N.A.	N.A.	N.A.	1/week for 6 weeks	Connaught	52.5 (N.A.)	7/20 (35%)	17/20 (85%)	18/20 (90%)	20/20 (100%)	6/20 (30%)	15/20 (75%)	14/20 (70%)
Ovesen et al. (1993) [11]	+	10	10/10 (100%)	N.A.	N.A.	N.A.	1/week for 6 weeks in 8/10, for 12 weeks in 2/10	Danish	26 (3–68)	N.A.	N.A.	8/10 (80%)	9/10 (90%)	N.A.	N.A.	10/10 (100%)
Bretton et al. (1989) [12]	+	23	23/23 (100%)	19/23 (83%)	4/23 (17%)	0/23 (0%)	1/week for 6 weeks	Pasteur	51.6 (6–105)	N.A.	13/23 (57%)	23/23 (100%)	23/23 (100%)	13/23 (57%)	13/23 (57%)	19/22 (91%)
Orihuela et al. (1989) [13]	–8/15+ 7/15	15	N.A.	N.A.	N.A.	N.A.	1/week for 6 weeks	Pasteur	N.A. (18–51)	14/15 (93%)	15/15 (100%)	13/15 (86%)	14/15 (93%)	13/15 (86%)	14/15 (93%)	15/15 (100%)
Palou et al. (1996) [14]	–17/18+ 1/18	18	15/18 (79%)	N.A.	N.A.	N.A.	1/week for 6 weeks	Connaught	31.1 (7–57)	17/18 (94%)	17/18 (94%)	13/18 (68%)	17/18 (94%)	15/18 (79%)	15/18 (79%)	17/18 (94%)
Taylor et al. (2007) [15]		28	28/28 (100%)	N.A.	N.A.	N.A.	1/week for 6 weeks in most cases	N.A.	90 (N.A.)	N.A.	20/28 (71%)	25/28 (89%)	25/28 (89%)	N.A.	19/28 (68%)	25/28 (89%)
Palou et al. (2006) [16]		11	6/11 (54%)	11/11 (100%)	0/11 (0%)	0/11 (0%)	1/week for 6 weeks in 10/11, for 12 weeks in 1/11	Connaught	40 (8–157)	7/11 (63%)	10/11 (90%)	9/11 (81%)	9/11 (81%)	6/11 (54%)	8/11 (72%)	11/11 (100%)
Canda et al. (2004) [17]		12	12/12 (100%)	7/12 (58%)	N.A.	5/12 (42%)	1/week for 6 weeks in 10/12, for 12 weeks in 2/12	Connaught	62.5 (24–110)	6/12 (50%)	8/12 (66%)	10/12 (83%)	11/12 (91%)	5/12 (42%)	7/12 (58%)	8/12 (66%)
Schellhammer et al. (1995) [18]	-	17	8/17 (47%)	N.A.	N.A.	N.A.	1/week for 6 weeks	Pasteur	64.0 (29–90)	9/17 (52%)	14/17 (82%)	11/17 (65%)	14/17 (82%)	8/17 (47%)	10/17 (59%)	16/17 (94%)
Hillyard et al. (1988) [19]	-	8	8/8 (100%)	N.A.	N.A.	N.A.	1/week for 6 weeks	Pasteur	22.3 (15–52)	6/8 (75%)	7/8 (86%)	5/8 (63%)	6/8 (75%)	N.A.	5/8 (63%)	8/8 (100%)

Almost two-thirds of patients who did not have TURP prior to BCG therapy had no recurrence (DFS 63–89%) in the prostatic urethra during follow up, and at least three out of four of these patients did not experience tumor progression to the prostate during follow up (PFS 75–91%). In four out of ten studies, only patients with primary urethral carcinoma were included. Only three out of ten studies differentiated between tumor stages, i.e. Tis pu versus Tis pd versus T1. The results of these studies were recorded and presented in different ways, which made it impossible to assess the different response rates of BCG therapy in the different tumor stages of PUC. In addition, the number of cases in the studies would have been too small to obtain reliable prognostic information. A study by Ovesen et al. [11] examined the Danish BCG strain and not the commonly used Pasteur or Connaught BCG strains. In the study by Taylor et al. [15] the strain used was not outlined.

Only in one study did 5 of 12 patients have stage T1 urethral carcinoma of the prostate [17]. In this study, the DFS (83%) and the PFS (91%) in the prostate were within the range of the remaining nine studies. However, the overall DSS was only 66% compared to an overall DSS of 70–100% in the other studies. It could not be completely excluded that these stromal invasive cases reduced the survival of the patients in this study compared to the other studies. Without access to the full data, we could not perform statistical analyzes.

### The role of TURP before BCG in prostatic urethral carcinoma

Prospective randomized clinical trials or even directly applicable clinical studies of good quality addressing the question if performing TURP prior to BCG improves DSS or lowers morbidity without compromising DSS in PUC were not available. This issue was exclusively addressed by retrospective cohort studies. Unfortunately, the authors of the selected studies did not use the same format in presenting their data. Some authors only described the results in the text, others used tables with limited information on e.g. follow-up, DFS, PFS or DSS.

A total of ten articles found that examined the effectiveness of BCG in urethral carcinoma in the prostatic urethra with or without TURP prior to BCG treatment (Table 2). The studies by Gofrit [10], Ovesen [11] and Bretton [12] only included patients who had been treated with TURP before BCG. Orihuela [13] and Palou [14] included both patients with and without TURP prior to BCG treatment.

Although an appropriate comparative statistical analysis is not suitable for these heterogeneously designed studies, there appears to be a pattern that suggests slightly better local response with higher DFS (80–100% vs. 63–89%) and PFS (90–100% vs. 75–94%) in the prostatic urethra in the studies in which patients were treated with TURP prior to BCG treatment compared to the studies in which no TURP was performed. However, this difference in recurrence and progression in the prostate affected neither the total PFS (57–75% vs. 58–93%) nor the DSS (70–100% vs. 66–100%).

## DISCUSSION

Intravesical BCG treatment with or without TURP prior BCG induction therapy was retrospectively evaluated in non-invasive stages of PUC. Upon reviewing the published literature, it becomes clear that the incidence of primary PUC is likely to be underestimated: Almost all cases of primary PUC were detected during the workup of primary bladder cancer or UC of the UUT. This impression is supported by the study by Giannarini et al, which indicated that an undiagnosed PUC could often be the cause of BCG failure. [21]. In this study, bladder CIS was an independent risk factor for BCG failure due to recurrence in the prostatic urethra or UUT. Since not every patient in this study had a TUR biopsy before starting BCG therapy, it was discussed that the actual incidence of PUC is difficult to estimate and is likely to be underestimated.

The most accurate method of detecting PUC during the primary workup of suspected urothelial cancer in the prostate is to perform a TUR biopsy, which has been shown to be superior to prostate needle biopsy and fine needle aspiration of the prostate [22]. Donat and Herr suggested a resectoscope loop biopsy of the prostatic urethra between the 5 and 7 o’clock positions from the bladder neck and distally around the verumontanum in patients with suspected PUC [23]. However, not only should suspicious lesions in the prostatic urethra be biopsied: Palou showed that almost 12% of patients with T1G3 bladder cancer had carcinoma in situ of the prostatic urethra on a resection biopsy [24]. In several series for the evaluation of cystectomy specimens, involvement of the prostatic urethra was found in 15–48% [25–27]. Both CIS in the bladder and multifocal tumor growth were associated with a higher risk of involvement of carcinoma of the prostatic urethra. With the data available, it seems reasonable to recommend resection biopsies in patients with positive urinary cytology of unknown origin, if CIS in the bladder or upper urinary tract is suspected, if bladder cancer is multifocal or occurs around the bladder neck, and when radical cystectomy is pending especially if chemotherapy is planned before surgery [6]. The AUA/SUO guidelines so far only recommend prostate loop biopsies in cases with a suspicious lesion in the prostate and in patients with a history of NMIBC with normal cystoscopy and positive cytology [5].

It was discussed that cases of PUC with stage T1 and Tis pd might have a worse prognosis than tumors with stage Tis pu [28]. There are no prospective studies on the conservative treatment of non-invasive PUC in its various tumor stages, which is why this hypothesis has not yet been confirmed. In seven of the ten available studies in this review, no TNM staging of PUC was carried out; and only one of the ten studies [17] included patients with stage T1 urethral carcinoma (*n* = 5). Accurate staging of tumors is the basic requirement for evaluating the prognostic effects of the extent of TURP prior to BCG treatment in malignant diseases of the urothelium of the prostatic urethra and ducts. This underscores the importance of improving the use of the current TNM staging system for PUC in the future.

According to the EAU-guidelines, a urethra-sparing approach with TURP and intravesical-BCG is indicated in patients with non-invasive UC or carcinoma in situ of the prostatic urethra and ducts (level of evidence 3, grade of recommendation C) [6]. In the available studies, of all BCG-treated patients who were not treated with TURP, almost 2/3 of the patients had no tumor recurrence in the prostate and at least 3/4 had no tumor progression in the prostate. In the available studies examining the use of TURP before BCG therapy, about 4/5 of the patients had no recurrence and 9/10 no tumor progression in the prostate. However, these improved local response rates after TURP had no influence on the overall response (PFS, DSS) in these cohorts. This finding supports the rationale for performing TURP prior to BCG treatment for non-invasive PUC, namely improving effectiveness of BCG treatment, at least with regard to the local reaction in the prostate, without necessarily improving the overall prognosis. Prospective studies need to confirm these results. In particular, the treatment of the rare cases of stage T1 PUC should be carefully studied.

Systemic side effects are seen in approximately 30% of patients treated with BCG for bladder cancer. Local symptoms of BCG instillations are cystitis, epididymitis or granulomatous prostatitis with dysuria, urination problems and hematuria. These symptoms occur in about 60% of BCG treated bladder cancer patients [29]. Approximately 70% of bladder cancer patients treated with BCG have both systemic and local side effects. In 16–22% of treated patients BCG treatment is discontinued because of toxicity [30]. Since almost all patients with PUC also have concomitant bladder cancer, specific side effects of BCG in the treatment of PUC could not be determined and cannot be found in the literature. It was therefore impossible to derive specific side effects of BCG therapy for PUC, which is a limitation of the study.

Apart from that, several other limitations of the current study have to be mentioned: Different definitions of progression were used in the cited studies. A comparative statistical analysis was not possible. No data on the natural history of the various non-invasive stages of PUC were available for comparison. Most studies only offered a BCG induction course. It cannot be ruled out that maintenance therapy for non-invasive PUC could have an improved prognosis. In addition, different BCG strains were used (Connaught and Pasteur). In one study [11] the rare Danish BCG strain was used. It is uncertain how this affected the results. Another problem is inconsistent follow-up of tumor status in the prostatic urethra: In some studies, cold cup biopsies were used more or less routinely, and in other studies, TUR biopsies of the prostate were taken only when there were visible or suspicious lesions. These differences in follow up schemes and methods might have affected differences of the outcomes after BCG therapy significantly.

## CONCLUSIONS

Taken together, the use of TUR biopsies of the prostatic urethra in appropriate cases during the primary work-up for suspected urothelial cancer, as well as the use of the current TNM classification for PUC, need to be improved. BCG therapy for non-stromal invasive stages of PUC show a good local response. Local response rates are further improved by a TURP, although the overall prognosis does not seem to be affected. Further evidence for BCG treatment in the rare cases of stromal invasive PUC is needed. Specific side effects of BCG treatment for PUC, either with or without TURP before treatment, are not reported.
